# Estimating Maize Leaf Water Content Using Machine Learning with Diverse Multispectral Image Features

**DOI:** 10.3390/plants14060973

**Published:** 2025-03-20

**Authors:** Yuchen Wang, Jianliang Wang, Jiayue Li, Jiacheng Wang, Hanzeyu Xu, Tao Liu, Juan Wang

**Affiliations:** 1 College of Hydraaulic Science and Engineering, Yangzhou University, Yangzhou 225009, China; 223203124@stu.yzu.edu.cn (Y.W.); wangjuan@yzu.edu.cn (J.W.); 2 Jiangsu Key Laboratory of Crop Genetics and Physiology/Jiangsu Key Laboratory of Crop Cultivation and Physiology, Agricultural College of Yangzhou University, Yangzhou 225009, China; dx120240146@stu.yzu.edu.cn (J.W.); 241604212@stu.yzu.edu.cn (J.L.); xuhanzeyu@yzu.edu.cn (H.X.); 3 Jiangsu Co-Innovation Center for Modern Production Technology of Grain Crops, Yangzhou University, Yangzhou 225009, China

**Keywords:** UAV, multispectral image, machine learning, wheat, leaf water content

## Abstract

Leaf water content (LWC) is a key physiological parameter for assessing maize moisture status, with direct implications for crop growth and yield. Accurate LWC estimation is essential for water resource management and precision agriculture. This study introduces a high-precision method for estimating maize LWC utilizing UAV-based multispectral imagery combined with a Random Forest Regression (RFR) model. By extracting vegetation indices, image coverage, and texture features and integrating them with ground-truth data, the study examines the variation in LWC estimation accuracy across different growth stages. The results indicate that the RFR model performs optimally during the seedling stage, with a root relative mean square error (RRMSE) of 2.99%, whereas estimation errors are larger during the tasseling stage, with an RRMSE of 4.13%. Moreover, the RFR model consistently outperforms multiple linear regression (MLR) and ridge regression (RR) models throughout the growing season, demonstrating lower errors on both training and testing datasets. Notably, the RFR model exhibits significantly reduced errors in the training dataset compared to both MLR and RR models. Following particle swarm optimization (PSO), the prediction accuracy of the RFR model is notably enhanced, with the RRMSE on the training dataset decreasing from 1.46% to 1.19%. This study provides an effective approach for estimating maize LWC across different growth stages, supporting crop water management and precision agriculture, and offering valuable insights for the estimation of water content in other crops.

## 1. Introduction

The moisture status of plants is widely regarded as a critical indicator of plant health, with direct implications for growth, development, and physiological function [1]. Water serves not only as the basis for cellular expansion and normal metabolic processes but also plays a pivotal role in photosynthesis, nutrient uptake, and overall physiological regulation [2]. In the growth cycle of crops such as maize, water is a determining factor in biomass production, leaf function, and ultimately, yield. Leaf water content (LWC), an essential parameter for assessing plant moisture status, reflects the water supply demandequilibrium of the crop. Under water deficit conditions, plant roots are unable to efficiently absorb water and nutrients, leading to restricted leaf transpiration, which in turn impairs photosynthesis, causing slow growth and, in severe cases, wilting and leaf chlorosis [3]. Excessive water, conversely, can induce oxygen deficiency in the roots, promoting root rot and other diseases that compromise plant health [4]. Consequently, the timely and accurate monitoring of maize leaf moisture is crucial for optimizing irrigation management, enhancing water use efficiency, and safeguarding crop yield [5].

At present, moisture monitoring in crop cultivation predominantly relies on traditional methods, such as manual sampling followed by drying and weighing to determine plant moisture content [6]. Although this approach can yield relatively accurate data, it is labor-intensive, time-consuming, and fraught with limitations. Manual sampling and measurement demand significant human resources, and the data acquisition process is relatively slow, resulting in poor timeliness and insufficient support for real-time monitoring [7]. With the rapid advancement of technology, agricultural practices are progressively shifting toward greater automation and precision. The limitations of conventional moisture monitoring techniques are increasingly apparent, creating a pressing need for more efficient, precise, and real-time monitoring technologies [8].

Remote sensing technology is a comprehensive technique that uses various sensors to collect, process, and ultimately image electromagnetic wave information radiated and reflected by distant targets, thereby detecting and identifying various features on the ground [9]. Remote sensing technology is primarily divided into satellite remote sensing and UAV (unmanned aerial vehicle) remote sensing [10]. Both of these remote sensing technologies are widely used in agriculture today. Zhang et al. [11], by analyzing HJ-CCD images obtained through satellite remote sensing and employing partial least squares (PLS), developed a model for estimating wheat yield, providing a more accurate and effective method for improving winter wheat yield estimation precision. Jeong et al. [12] incorporated data from UAV remote sensing into the GRAMI model, achieving precise predictions of rice yield. While UAV remote sensing systems cover a smaller area and have a shorter monitoring period compared to satellite systems, they offer advantages such as lower cost, easier operation, greater flexibility in application scenarios, and higher image resolution (with pixel sizes smaller than 1 cm) [13]. These advantages overcome various issues present in satellite remote sensing systems in precision agriculture, making UAV systems more suitable for field reconnaissance [14].

When plants are subjected to varying moisture conditions, differences in tissue structure, physiological and ecological indicators, and morphology arise, leading to distinct reflectance spectral characteristics based on these changes [15]. This variation supports the feasibility of utilizing remote sensing technology to assess plant moisture status. Extensive research has been conducted on the application of UAV-based multispectral imagery for estimating crop moisture content. Wang et al. [16] employed remote sensing to measure the hyperspectral reflectance, chlorophyll content, and leaf area index of maize leaves, analyzing the relationship between canopy reflectance and canopy moisture content under different moisture stress levels. They developed a model capable of accurately estimating spring wheat canopy moisture stress. Chen et al. [17] used a six-band multispectral camera embedded in a UAV to capture images of cotton during the flowering and boll-forming stages. By identifying sensitive spectral bands associated with different parts of the cotton plant, they developed a model that more effectively monitors cotton plant moisture content. Yang et al. [18], using UAV multispectral imagery, applied a stacked ensemble model to more precisely determine the relationship between moisture content and the moisture stress experienced by winter wheat.

Maize, as one of the most important global crops, is widely utilized in food, energy, animal feed, and other industries, making it an integral part of the global agricultural production system [19]. Its high yield and efficiency position it as a cornerstone crop in the global food supply chain. The moisture content of maize also influences its physiological characteristics and health status. When water-stressed, changes in the maize plant occur, such as alterations in leaf color, structure, and texture, including wrinkling, discoloration, and yellowing [20]. The application of UAV multispectral imagery has provided new approaches for monitoring maize moisture. Shu et al. [19] used canopy coverage data derived from UAV hyperspectral imagery as a substitute for the maize leaf area index in big data models, enabling rapid monitoring of maize moisture status. Ndlovu et al. [21], using UAV multispectral data, applied various big data models to estimate maize moisture content and compared these estimates with actual measurements, identifying a model capable of accurately predicting maize moisture content. Niu et al. [22] developed an FVC model, incorporating five vegetation indices and three regression algorithms based on UAV multispectral imagery, which is applicable to various growth seasons, developmental stages, and crop moisture stress conditions, thus elucidating the relationship between maize moisture content and vegetation cover.

Although significant progress has been made in the use of UAV multispectral imagery for monitoring maize LWC, there are still limitations in the accuracy of model applications. Traditional linear regression methods exhibit constraints during model fitting, resulting in relatively low accuracy [23]. With the rapid advancement of artificial intelligence technologies, machine learning and deep learning algorithms are increasingly being applied to agricultural models. Algorithms such as multiple linear regression (MLR), ridge regression (RR), random forest regression (RFR), and particle swarm optimization (PSO) have demonstrated excellent performance in the inversion of crop physiological and biochemical parameters, providing high estimation accuracy. This study leverages these advanced machine learning and optimization algorithms to develop an efficient model for estimating maize LWC by integrating multiple multispectral image features with machine learning techniques. The specific research objectives are as follows: (1) To explore methods for estimating maize leaf moisture content at different growth stages, utilizing UAV multispectral imagery along with various spectral indices and texture features to enhance estimation accuracy; (2) To evaluate and compare the performance of machine learning algorithms, assessing the effectiveness of MLR, RR, and RFR in estimating maize leaf moisture content and identifying the optimal algorithm; (3) To enhance model performance through optimization techniques such as PSO, thereby improving the accuracy and reliability of LWC estimation. Ultimately, this study aims to provide an accurate and reliable method for estimating maize leaf water content and offer insights for the estimation of other agronomic parameters in crops, as well as for the application of multispectral UAVs in agricultural research.

## 2. Materials and Methods

### 2.1. Study Area and Experimental Design

**Figure 1:**
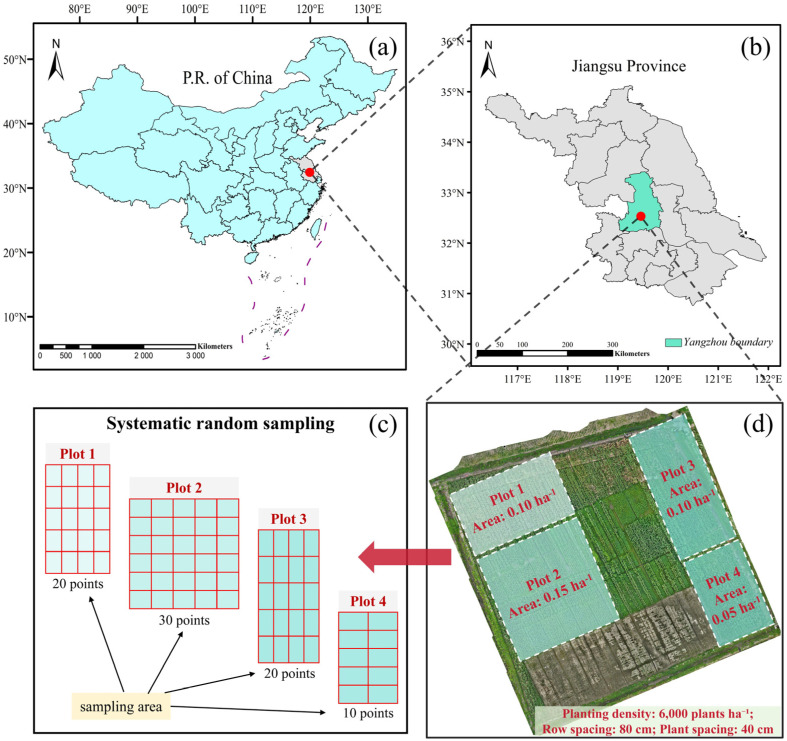
Research sites and experimental field layout. Note, (**a**) Map of China; (**b**) gray, map of Jiangsu Province; green, boundary of Yangzhou; (**c**) schematic diagram of the systematic random sampling; (**d**) orthographic projection of the test site and plot area distribution.

This study was conducted from May to July 2024 at the Jiang Wang Experimental Field of Yangzhou University, located in the Hanjiang District, Yangzhou City, Jiangsu Province, China (119°20′ E, 32°22′ N) (Figure 1). The region is characterized by a subtropical monsoon humid climate, with an average annual sunshine duration of 2140 h, annual precipitation of 1020 mm, and an average annual temperature of 17.5 °C. The physicochemical properties of the soil in the experimental area are as follows: bulk density of 1.38 g cm^−3^, pH of 8.1, total nitrogen content of 0.72 g kg^−1^, alkali-hydrolyzable nitrogen content of 67.3 mg kg^−1^, available potassium content of 43.1 mg kg^−1^, available phosphorus content of 14.8 mg kg^−1^, and organic matter content of 12 g kg^−1^.

The experiment followed a randomized complete block design (RCBD) with the maize variety *SuyuNuo11*. The planting density in the field was set at 6000 plants per hectare, with row and plant spacings of 80 cm and 40 cm, respectively. The experimental area was divided into four blocks, with three replicates per block to ensure the reliability of the data and the scientific validity of the results. At sowing, a conventional compound fertilizer was applied at a rate of 75 kg N·ha^−1^, and at the six-leaf stage, 150 kg N·ha^−1^ of urea was applied as top-dressing. Rainfed irrigation was employed throughout the experiment to ensure uniformity in fertilization and water supply. Data collection was conducted across three critical maize growth stages: seedling (BBCH 12), jointing (BBCH 31), and booting (BBCH 45) to evaluate the effects of varying planting densities and spacings on maize growth. The BBCH scale is an internationally standardized system for crop growth staging, providing a uniform framework for defining plant developmental phases. In this study, BBCH 12, BBCH 31, and BBCH 45 correspond to seedling establishment, rapid stem elongation, and the early reproductive stage, respectively. These phases represent key transitions from vegetative to reproductive growth, significantly influencing plant architecture and yield formation.

### 2.2. Data Collection

#### 2.2.1. Image Data Acquisition

The DJI Mavic 3 Multispectral UAV (Shenzhen Dajiang Innovation Technology Co., Ltd., Shenzhen, China) was employed to capture maize canopy leaf imagery within the experimental area. Data collection occurred between 10:00 a.m. and 2:00 p.m. to ensure that the solar altitude angle remained above 30°, thus mitigating the impact of low-light conditions on image quality. The multispectral imaging system was equipped with four monochromatic sensors: Green (G, 560 nm ± 16 nm), Red (R, 650 nm ± 16 nm), Red Edge (RE, 730 nm ± 16 nm), and Near-Infrared (NIR, 860 nm ± 26 nm). The camera featured a 5-megapixel 1/2.8-inch CMOS image sensor with an equivalent focal length of 25 mm and a field of view of 73.91°. The UAV was outfitted with an RTK module to achieve centimeter-level positioning accuracy, ensuring the precise georeferencing of the images.

The mission was conducted at a flight altitude of 20 m, at a speed of 1.7 m/s. The lateral and longitudinal overlaps were 70% and 80%, respectively, ensuring comprehensive coverage and image consistency. Data collection took place during three distinct maize growth stages on the following dates: 15 May 2024 (seedling stage), 14 June 2024 (jointing stage), and 8 July 2024 (booting stage). The ground sample distance (GSD) of the multispectral imagery was 0.92 cm per pixel. The flight duration was 15 min and 1 s, during which a total of 451 images were acquired.

#### 2.2.2. Field Measured Data Acquisition

Field data collection was conducted across the three maize growth stages and carried out on the same day as UAV image acquisition to ensure data consistency and timeliness. A total of 80 samples were collected at each growth stage, amounting to 240 samples in total. To ensure spatial representativeness, a systematic random sampling approach was adopted, with sample allocation determined based on the proportional area of each plot. Specifically, 20, 30, 20, and 10 samples were collected from plots 1, 2, 3, and 4, respectively. For implementation, sampling points were evenly distributed within each plot, following a predefined grid interval to ensure comprehensive spatial coverage. Additionally, a randomly selected starting point was used to mitigate potential human bias. The spatial distribution of the samples is illustrated in Figure 1c.

Each sample was weighed in the field to obtain its fresh weight (g), after which it was placed in a labeled envelope for identification. All samples were sent to the laboratory for processing, where they were first blanched at 105 °C for 30 min. The temperature was then reduced to 80 °C, and the samples were dried to a constant weight. Finally, the dry weight (g) of each sample was recorded. Data organization and analysis were performed using Office Excel 2024 (Microsoft, Redmond, WA, USA).

### 2.3. Data Preprocessing

**Figure 2:**
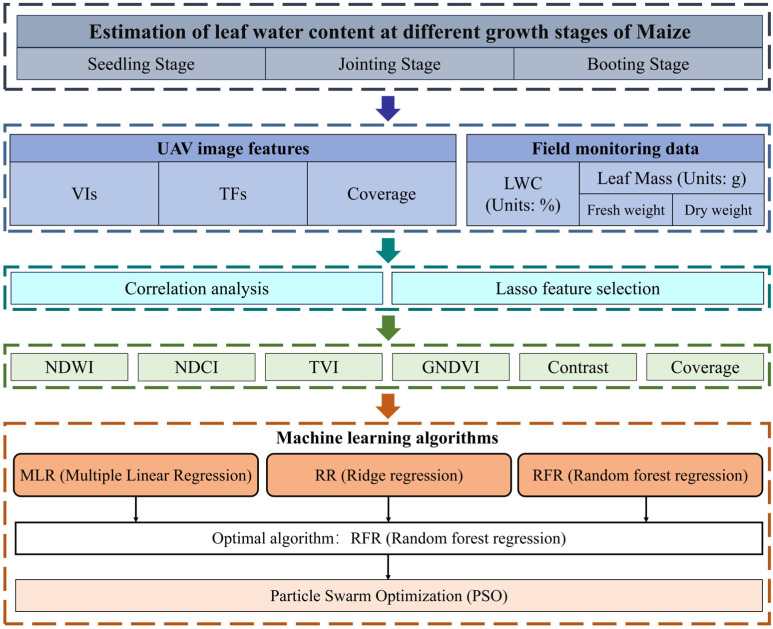
Flowchart of the experiment methodology.

Image preprocessing was performed following data collection, utilizing DJI Terra (Shenzhen Dajiang Innovation Technology Co., Ltd., Shenzhen, China), ENVI 5.6 (Exelis Visual Information Solutions, Boulder, CO, USA), and ArcGIS 10.8 (Esri Corporation, Redlands, CA, USA) software. The preprocessing procedure included the following steps (see Figure 2): (a) image import, (b) radiometric calibration, (c) image reconstruction, (d) image fusion, and (e) subplot clipping.

The procedure is as follows: After importing the UAV-collected image data from the storage device into DJI Terra V4.4.6 software, radiometric calibration is performed using three standard gray gradients (25%, 50%, 75%) to derive true reflectance data and generate orthophotos. The radiometrically corrected two-dimensional reconstruction layer is then fused in ENVI 5.6 based on the wavelength sequence (Green < Red < Red-Edge < NIR) to construct the multispectral image. Using the editing tools in ArcGIS 10.8, segmentation is first carried out for the entire experimental area, followed by a refined segmentation based on the locations of the ground sampling points. Python 3.10 algorithms (Guido van Rossum, 1998) are employed to perform clipping according to the Shpfile.

### 2.4. Feature Extraction

#### 2.4.1. Vegetation Index Extraction

Following whiteboard calibration, the Green (G), Red (R), Near-Infrared (NIR), and Red-Edge (RE) bands from the UAV-collected multispectral images are combined through linear or nonlinear methods to derive vegetation indices (VIs) for subsequent model development. Based on prior research, this study compiles 11 vegetation indices associated with maize water content (see Table 1).

#### 2.4.2. Texture Feature Extraction

Texture features, extracted through image processing techniques, are used to quantitatively or qualitatively characterize the texture structure of images. These features serve as critical indicators in target detection and image classification and are extensively applied in areas such as yield estimation. The Gray Level Co-occurrence Matrix (GLCM), introduced by Haralick et al. [34], generates a co-occurrence matrix by analyzing the relative positions of pixels within an image. This matrix represents the spatial gray-level dependencies between pixels, thereby reflecting the texture information of the image. While the GLCM provides valuable insights into the direction, interval, and range of image gray levels, it cannot directly describe texture characteristics. Instead, statistical properties must be employed to quantitatively define texture features. The five commonly used statistical properties for texture analysis are as follows:

(1) $$Contrast=\sum_{i}\sum_{j}p(i,j)*(i-j{)}^{2}$$

(2) $$Correlation=\sum_{i}\sum_{j}\frac{(i-mean)*(j-mean)*p(i,j{)}^{2}}{Variance}$$

(3) $$Energy=\sum_{i}\sum_{j}p\left(i,j\right)*\ln p\left(i,j\right)$$

(4) $$Homogeneity=\sum_{i}\sum_{j}p\left(i,j\right)*\frac{1}{1+(i-j{)}^{2}}$$

(5) $$ASM=\sum_{i}\sum_{j}p(i,j{)}^{2}$$

where p represents the gray level difference between two pixels, and i and j represent the pixel values of the gray level. The value of GLCM (i,j) represents the number of pixels in the image with pixel value i adjacent to pixel value j. The acronym ASM refers to Angular Second Moment.

#### 2.4.3. Image Coverage Features

Vegetation coverage refers to the proportion of area or spatial extent occupied by a particular crop within a given region [35]. This metric is used to describe both the relative significance of the crop in surface coverage and its distribution characteristics. In remote sensing, vegetation coverage is a vital tool for analyzing and monitoring vegetation conditions in imagery [24], as it allows for precise extraction and quantification of the distribution and temporal changes in surface vegetation. This study employs a threshold-based image segmentation technique to estimate the vegetation coverage of the wheat canopy. By setting an appropriate threshold, this method distinguishes between green vegetation pixels and background pixels, thus enabling the calculation of the proportion of vegetation coverage in the image. The formula for computing vegetation coverage is as follows:

(6) $$\begin{array}{c} Coverage=\frac{G}{N} \end{array}$$

where G represents the number of pixels identified as green vegetation in the image, and N represents the total number of non-NaN (i.e., not undefined) pixels in the image.

### 2.5. Machine Learning Algorithms

This study utilizes three widely used machine learning algorithms—multiple linear regression (MLR), ridge regression (RR), and random forest regression (RFR)—to estimate the leaf water content (LWC) of maize. The process for implementing these machine learning algorithms is outlined in the following steps:

(1) All image features and leaf water content data are normalized;
(2) The dataset is randomly divided into two subsets, with 50% allocated to the training set and the remaining 50% to the test set;
(3) Ten-fold cross-validation is applied to the training set. By performing multiple iterations of training and validation, the variability introduced by data partitioning is minimized, allowing for a more accurate evaluation of the model’s generalization ability and a reduction in overfitting risk. Finally, the optimal model is selected, and its performance in estimating leaf water content is assessed using the test set.

#### 2.5.1. MLR Algorithm

MLR is a classical statistical technique that establishes linear relationships between multiple independent variables and a dependent variable for data analysis and prediction [36]. MLR assumes that variations in the dependent variable are influenced by the combined effects of multiple independent variables. By optimizing the combination of independent variables, MLR can offer more accurate predictions or estimations of the dependent variable. In this study, the MLR algorithm is employed to estimate the leaf water content of maize, implemented using Python 3.10. This approach effectively estimates the leaf water content based on the relationships between various spectral features and water.

#### 2.5.2. RR Algorithm

RR is an enhanced regression analysis method designed to address the issue of multicollinearity. Unlike ordinary least squares (OLS), ridge regression incorporates an L2 regularization term in the regression model, which penalizes the regression coefficients to reduce model complexity and prevent overfitting [37]. The regularization term enables ridge regression to maintain model stability and predictive accuracy, even in the presence of multicollinearity. In this study, ridge regression is employed to model the relationship between multiple spectral features and the leaf water content of maize, with model training and testing conducted using Python 3.10 and the Scikit-learn library. By fine-tuning the regularization parameter, the optimal model is identified, and the RR model demonstrates exceptional predictive stability in both cross-validation and test set evaluations.

#### 2.5.3. RFR Algorithm

RFR is an ensemble learning algorithm that performs regression tasks by aggregating multiple decision trees, demonstrating robust generalization capabilities and resistance to overfitting [38]. The RFR model employs bootstrapping and bagging techniques to generate several decision trees and then combines the predictions from these trees to derive the final regression outcome through either voting or averaging. In this study, RFR is used to estimate the water content of maize leaves by constructing multiple decision trees and combining them into a potent regression model. The RFR model effectively leverages randomly selected subsets of image features and sample subsets, integrating the predictions from various trees to improve both the stability and accuracy of the model.

#### 2.5.4. Particle Swarm Optimization Algorithm (PSO)

**Figure 3:**
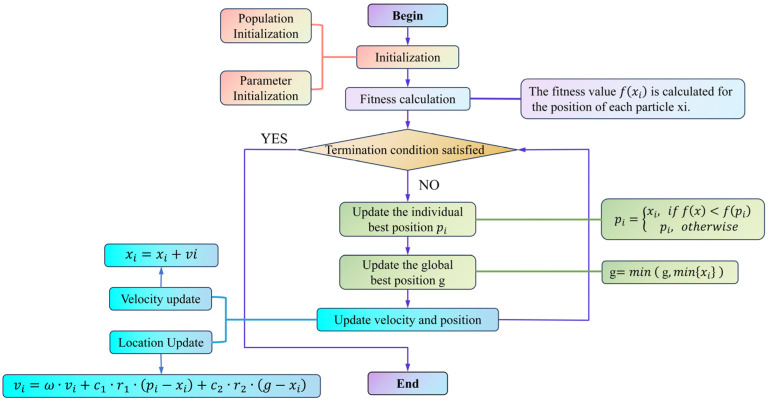
Schematic structure of the PSO optimization algorithm.

The PSO algorithm, introduced by Kennedy and Eberhart in 1995, is a population-based stochastic optimization method widely utilized for continuous optimization problems [39]. Inspired by the collective behavior of bird flocks and fish schools, PSO enables each particle to iteratively update its velocity and position based on both its individual experience and that of other particles, progressively converging toward the global optimum. By leveraging personal best (pbest) and global best (gbest) mechanisms, PSO maintains a balance between exploration and exploitation, thereby enhancing search efficiency. A schematic representation of the PSO algorithm is provided in Figure 3.

**Figure 4:**
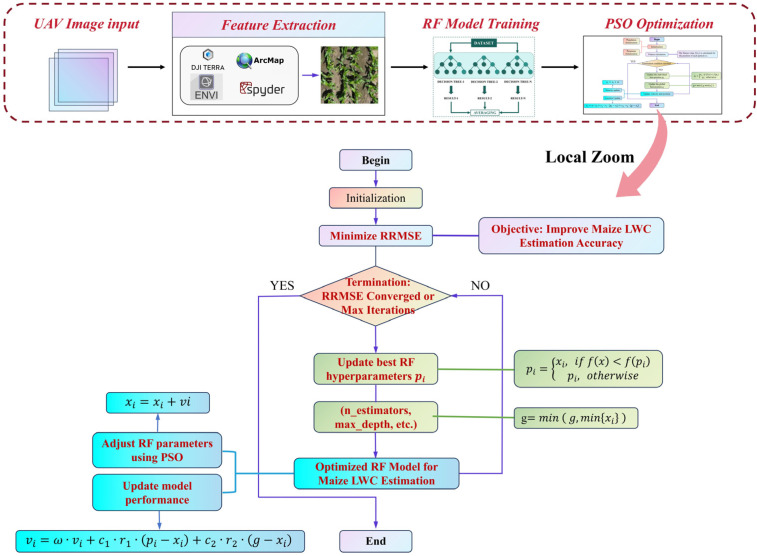
Workflow of PSO optimization for enhancing RF model performance in maize LWC estimation.

The PSO optimization workflow in this study is depicted in Figure 4, comprising key steps such as UAV image acquisition, feature extraction, RFR training, and PSO-based hyperparameter tuning to ensure systematic and reproducible model optimization [40]. PSO is employed specifically to optimize the hyperparameters of the RFR model, thereby enhancing the accuracy and stability of maize LWC estimation. The optimization objective is to minimize the relative root mean square error (RRMSE), improving the model’s generalization capability. In this study, PSO initializes a population of particles, each representing a potential hyperparameter combination, which iteratively explores the search space to identify the optimal solution. The optimized hyperparameters include the number of decision trees (n_estimators: 50–500, step size 10), maximum tree depth (max_depth: 5–50, step size 5), and minimum samples required for a split (min_samples_split: 2–10, step size 1). During each iteration, the velocity and position of the particles are updated according to the following equations:

(7) $$v_{i}=\omega \cdot v_{i}+c_{1}\cdot r_{1}\cdot \left(p_{i}-x_{i}\right)+c_{2}\cdot r_{2}\cdot \left(g-x_{i}\right)$$

(8) $$x_{i}=x_{i}+v_{i}$$

where ω represents the inertia weight, initially set to 0.9 and gradually decreasing to 0.4. $c_{1}$ and $c_{2}$ are learning factors, both set to 2.0, while $r_{1}$ and $r_{2}$ are random numbers within the range of 0 to 1. $p_{i}$ denotes the individual best solution, and g represents the current global best solution. Throughout the optimization process, the fitness value of each particle is determined based on the RRMSE predicted by the RFR model (Equation 8). If a newly generated parameter combination yields an RRMSE lower than the current best value, pbest and gbest are updated; otherwise, they remain unchanged. The optimization continues until one of the termination criteria is met: PSO terminates when the RRMSE change remains below 0.01% for 10 consecutive iterations or when the maximum iteration limit (50) is reached, ensuring the identification of the optimal hyperparameter combination (Figure 4).

### 2.6. Evaluation Metrics

To thoroughly assess the model’s performance, this study employs commonly used evaluation metrics, including Relative Root Mean Square Error (RRMSE) and the coefficient of determination (R^2^), to quantify both the accuracy and stability of the model in estimating the leaf water content (LWC) of maize leaves. The formulas for these evaluation metrics are presented below:

(9) $$RRMSE=\frac{\sqrt{\frac{1}{n}\sum_{i=1}^{n}(y_{i}-{\hat{y}}_{i}{)}^{2}}}{\frac{1}{n}\sum_{i=1}^{n}y_{i}}$$

(10) $$R^{2}=\frac{\sum_{i=1}^{n}({\hat{y}}_{i}-{\bar{y}}_{i}{)}^{2}}{\sum_{i=1}^{n}(y_{i}-{\bar{y}}_{i}{)}^{2}}$$

where $y_{i}$ represents the true value of the sample target variable; ${\hat{y}}_{i}$ represents the predicted value; ${\bar{y}}_{i}$ represents the mean value; and n represents the number of samples.

## 3. Results

### 3.1. Descriptive Statistics of Maize LWC

This study quantitatively analyzed the LWC of maize leaves across different growth stages (Table 2). Data from three stages—germination, jointing, and booting—revealed significant dynamic changes in LWC, reflecting the plant’s evolving water requirements and regulatory mechanisms. During the germination stage, when seeds absorb considerable amounts of water, the leaf water content was generally higher. The maximum value observed was 88.2%, the minimum 69.6%, and the average 76.9%. The standard deviation (STDEV) was 2.27%, and the coefficient of variation (CV) was 3.14%, indicating low dispersion and strong regularity in water content fluctuations during this stage. While the data distribution was nearly symmetric, the skewness value of 0.34 suggested a slight tendency toward higher water content. In the jointing stage, LWC decreased, with the maximum value dropping to 72.6%, the minimum to 60.8%, and the average to 67.5%. The STDEV was 2.49%, and the CV was 3.46%, slightly higher than during germination, reflecting increased water content variability. The skewness value of 0.15 was close to zero. The decline in water content was attributed to reduced water absorption by the roots and increased leaf transpiration. During the booting stage, the plant’s water demand increased again, coinciding with rapid grain development, and the leaf water content showed a slight rebound. The maximum value in this stage was 80.3%, the minimum 60.9%, and the average 75.9%. The STDEV was 2.98%, and the CV was 4.16%, both higher than in the jointing stage, indicating more pronounced fluctuations in water content. The skewness was 0.20. This increase in water content reflects the plant’s heightened water demand, particularly during the critical period of nutrient transport and grain development. Despite the fluctuations in LWC across different growth stages, the CV remained relatively low—3.14%, 3.46%, and 4.16%, respectively—suggesting strong regularity in water content changes. The low dispersion also indicates the stability of water variation during each growth stage. The results from skewness and standard deviation further elucidate the distribution and fluctuation patterns of water content at different stages. In summary, the LWC of maize leaves exhibited a regular pattern of change throughout the growth stages, closely aligned with the plant’s physiological needs. These data not only confirm that the experimental design is consistent with the natural growth patterns of maize but also validate the accuracy and reliability of the experiment. Furthermore, these findings provide reliable data to support the development of future models for estimating maize water content.

### 3.2. Correlation Analysis of Features

**Figure 5:**
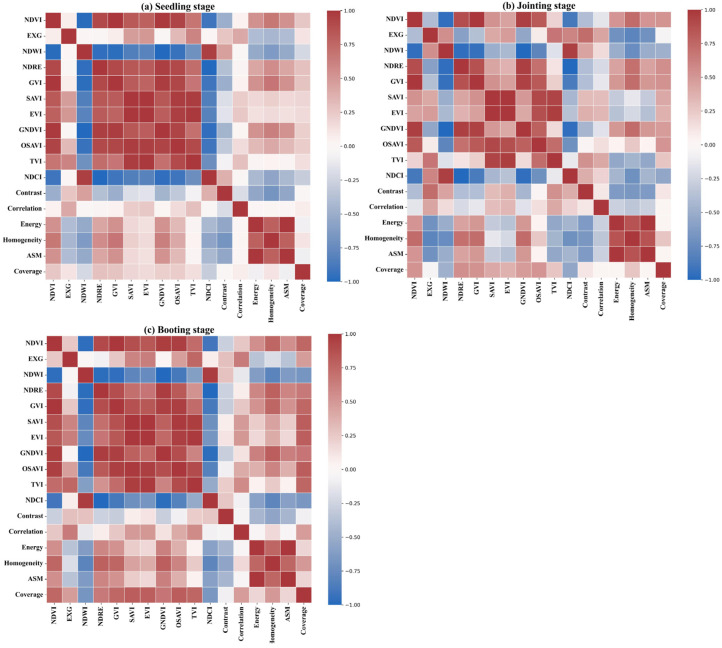
Correlation matrix of maize remote sensing features across different growth stages.

This study analyzed 17 conventional remote sensing features of maize. Due to significant correlations among these features, many provided redundant information, which not only reduced the model’s training efficiency but also increased computational complexity, potentially leading to overfitting and reduced generalization capacity. To address this, we first performed a Pearson correlation analysis (PCC) and created a correlation heatmap to identify the most representative features, minimizing the influence of redundancy (Figure 5). The correlation matrix revealed substantial redundancy between features across different growth stages (germination, jointing, and booting). During the germination stage (Figure 5a), a strong positive correlation (R > 0.80) was observed between NDVI, EXG, EVI, and GNDVI, while NDRE and GVI exhibited a strong negative correlation (R < −0.70). Specifically, the correlation between NDVI and EXG was 0.91, and between GNDVI and NDVI was 0.87, indicating substantial redundancy. Therefore, it is recommended to retain only one of these features in the model to reduce redundancy. In the jointing stage (Figure 5b), the correlations between NDVI, GVI, GNDVI, and EVI remained high, with coefficients exceeding 0.85. Notably, the correlation between NDVI and GVI was 0.91, and between GNDVI and EVI, it was 0.89. Additionally, the correlation between NDCI and Contrast was 0.78, further suggesting that these features convey similar information. Thus, one of these features could be selected for further analysis to streamline the feature set. In the booting stage (Figure 5c), feature correlations weakened, though redundancy was still evident. For instance, high correlations were found between NDVI, EXG, and GVI (R = 0.90, 0.85, 0.88), while NDRE and GNDVI exhibited a negative correlation (R = −0.74). These redundancies suggest that the features provide similar information on biomass variation, reinforcing the need to simplify the feature set. Overall, the correlation analysis across all growth stages demonstrated that NDVI, EXG, GVI, and GNDVI were strongly correlated, making them key indicators of plant growth and biomass changes. However, the high correlation between NDVI and both GVI and GNDVI (0.91 and 0.87, respectively) indicated redundancy across all stages. Therefore, in the feature selection process, it is essential to eliminate redundant features and retain those that effectively capture plant growth dynamics, thereby optimizing model inputs and enhancing analysis efficiency.

Through PCC redundancy analysis, we ultimately selected the following features, which are both highly representative and relatively independent: NDCI, TVI, NDWI, and Coverage. These features exhibited low correlation across growth stages and were effective in describing key changes during the plant’s growth process. Leveraging these non-redundant features, we can further refine the maize water content estimation model, thereby enhancing prediction accuracy.

### 3.3. Explained Variance of Features and Lasso Selection

**Figure 6:**
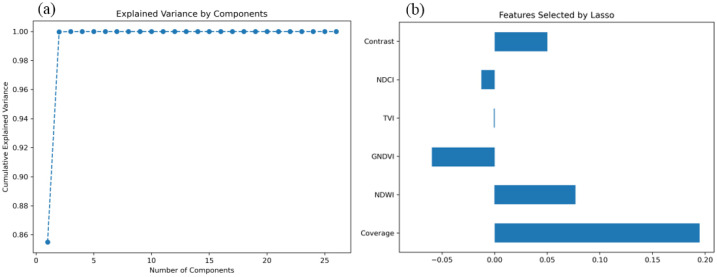
Explained variance and Lasso-selected features for maize leaf water content estimation.

Principal component analysis (PCA) was performed on all remote sensing features to evaluate the explained variance of each feature within the dataset. As shown in Figure 6a, the cumulative explained variance of the principal components indicates that the first two components account for the majority of the variance, with the cumulative explained variance approaching 1.00. This suggests that a small number of principal components effectively capture the primary variations in the data. This result highlights that a combination of multiple spectral features provides more comprehensive information for estimating maize leaf water content, while individual spectral indices are insufficient to independently represent water content fluctuations.

Building on the PCA results, feature selection was further conducted using the Lasso regression model, which employs L1 regularization to identify the most relevant features for maize leaf water content estimation. Figure 6b illustrates the selected features and their respective coefficients. The results indicate that six key features contribute substantially to the model: Coverage, NDWI (Normalized Difference Water Index), GNDVI (Green Normalized Difference Vegetation Index), TVI (Triangular Vegetation Index), Contrast, and NDCI (Normalized Difference Chlorophyll Index). As illustrated in Figure 6b, Coverage and NDWI exhibit the highest coefficients, at 0.195 and 0.078, respectively, underscoring their significance in the model. NDWI, a critical indicator of vegetation water content, demonstrates a strong contribution in Lasso regression, further confirming its essential role in water estimation. Coverage, which reflects vegetation density, is closely associated with water status, as areas with higher coverage generally indicate healthier vegetation and greater water availability, making it a crucial factor in LWC estimation. Contrast and GNDVI also exhibit notable contributions. GNDVI, which integrates green and near-infrared spectral bands, effectively captures vegetation chlorophyll content, which correlates with water status. Contrast, on the other hand, enhances monitoring accuracy by revealing the influence of water on leaf microstructure through texture analysis. Although NDCI and TVI contribute less than the aforementioned features, they still outperform other candidate variables. PCC analysis (Figure 5) indicates that these two features remain representative of LWC estimation across different growth stages and exhibit relative independence. Consequently, they were incorporated into the final model to enhance overall predictive capability.

In conclusion, this study employed the PCC + Lasso approach to select Coverage, NDWI, GNDVI, TVI, Contrast, and NDCI as modeling features, optimizing the model’s generalization ability and robustness.

### 3.4. Estimation Results of Maize LWC Across Various Growth Stages

**Figure 7:**
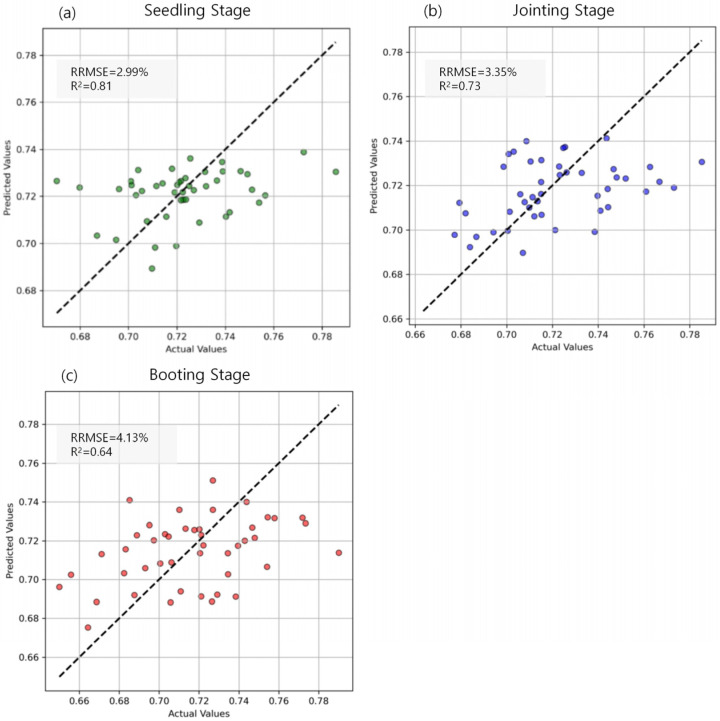
Prediction performance of maize leaf water content at different growth stages.

Using the PCC + Lasso method, this study identified Coverage, NDWI, GNDVI, TVI, Contrast, and NDCI as key features and employed RFR for modeling analysis. This approach aimed to improve estimation accuracy and evaluate the contributions of different variables to maize water content prediction. The regression analysis results (Figure 7) demonstrate that the predictive performance across the three growth stages is generally satisfactory; however, model error increases as the growth process progresses. Specifically, at the seedling stage, the RRMSE is 2.99%, with an R^2^ of 0.81 (Figure 7a). At the jointing stage, these values increase to 3.35% and 0.73, respectively (Figure 7b), and at the booting stage, they further rise to 4.13% and 0.64 (Figure 7c). These findings suggest that the RFR (Random Forest Regression) model exhibits high estimation accuracy across all growth stages, though its predictive precision is influenced by physiological traits and environmental factors. At the seedling stage, water content remains relatively stable, with most data points concentrated between 0.70 and 0.74. Under these conditions, the model effectively captures water content variations, achieving the lowest RRMSE (2.99%) and the highest R^2^ (0.81), indicating strong agreement between predicted and observed values. As maize enters the jointing stage, its water demand increases and growth accelerates, leading to more complex water distribution patterns. Additionally, external environmental factors such as precipitation and soil moisture exert a greater influence, resulting in an increase in RRMSE to 3.35% and a decrease in R^2^ to 0.73. Nevertheless, features selected through Lasso regression, such as Coverage and NDWI (Normalized Difference Water Index), continue to provide meaningful information, ensuring minimal deviation between predictions and actual values. At the booting stage, water demand rises sharply, particularly during grain development, leading to increased water fluctuations. Consequently, the RRMSE increases to 4.13%, while R^2^ declines further to 0.64. Despite the increased complexity of the prediction due to uneven water distribution and fluctuating environmental conditions, the model still successfully captured the major trends in water content changes. However, the significant water fluctuations led to an increase in prediction errors. Overall, while the RRMSE during the booting stage was higher compared to the other growth stages, the R^2^ and RRMSE results indicate that the RFR model consistently delivered stable predictions across all stages—seedling, jointing, and booting. By selecting features through the PCC and Lasso regression processes, the model effectively reduced redundancy and enhanced prediction accuracy. Specifically, the importance of Coverage and NDWI in the model was further confirmed, validating their essential role in estimating maize leaf water content.

### 3.5. Comparison of Estimation Results Using Different Regression Models

**Figure 8:**
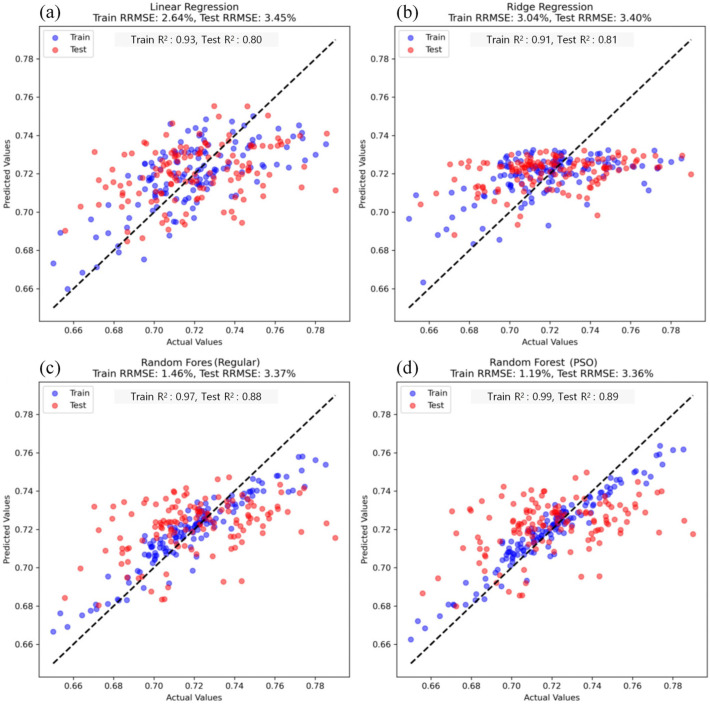
Comparison of model performance for maize leaf water content estimation.

This study evaluated the performance of three different models for predicting maize LWC (Figure 8): MLR, RR, and RFR. The results indicate that for the MLR model, the RRMSE was 2.64% with an R^2^ of 0.93 in the training set, while in the test set, these values were 3.45% and 0.80, respectively. This suggests that although the MLR model fits the data well during training, its generalization ability is relatively weak in the test phase, leading to higher errors. The RR model exhibited a training RRMSE of 3.04% and an R^2^ of 0.91, while the test RRMSE was 3.40% with an R^2^ of 0.81. Although this represents a marginal improvement, the error reduction compared to the MLR model was not substantial. In contrast, the RFR model demonstrated superior performance across both the training and testing phases. The training RRMSE was 1.46%, with an R^2^ of 0.97, while the test RRMSE was 3.37%, with an R^2^ of 0.88, significantly outperforming both the MLR and RR models. During training, the RFR model exhibited the lowest error, reducing it by 1.58% compared to the other models. Although the differences in test RRMSE between the RFR, MLR, and RR models were minor, the RFR model still maintained the lowest error, with a reduction of 0.08% compared to the other models. These findings indicate that the RFR model achieved the highest predictive accuracy and generalization ability in this study, making it the most effective model for estimating maize leaf water content.

To further enhance the predictive performance, the PSO algorithm was applied to optimize the RFR model. Following PSO optimization, the model’s accuracy improved, with the RRMSE of the training and test sets decreasing to 1.19% and 3.36%, respectively, while R^2^ increased to 0.99 (training set) and 0.89 (test set). The optimized model exhibited significantly reduced training errors and enhanced fitting capability, indicating that PSO effectively fine-tuned the hyperparameters, enabling the RFR model to better adapt to data distributions and improve prediction accuracy.

## 4. Discussion

This study employed multispectral UAV image data and three regression models (MLR, RR, and RFR) to estimate the water content of maize at various growth stages. The results indicate that the RFR achieved the best performance in water content prediction, with RRMSE of 1.46% for the training set and 3.37% for the test set. Following PSO, the model’s predictive accuracy improved further, with RRMSE values reduced to 1.19% for the training set and 3.36% for the test set. The subsequent discussion will address the model’s performance, feature selection, growth stage differences, and the effects of optimization.

### 4.1. Comparison of Model Performance for LWC Estimation

The three regression models employed in this study—MLR, RR, and RFR—demonstrated significant differences in their ability to estimate maize water content. The MLR model showed strong performance on the training set, with a training RRMSE of 2.64%. This suggests that MLR is effective when there is a linear relationship between the variables. However, its performance on the test set was suboptimal, with a test RRMSE of 3.45%. This highlights the main limitation of the MLR model: it assumes a linear relationship between the independent and dependent variables, yet maize leaf water content often exhibits a nonlinear relationship with multispectral features. As a result, the MLR model struggles to capture the underlying structure of complex, nonlinear data, leading to weak generalization and poor predictive performance. The RR model addressed multicollinearity by incorporating L2 regularization and reducing overfitting through model coefficient constraints. The training RRMSE was 3.04%, and the test RRMSE was 3.40%. While the RR model mitigated overfitting to some extent compared to the MLR model, it still failed to significantly improve the test set’s prediction accuracy. This suggests that, despite its ability to handle correlations between independent variables, the RR model’s linear assumptions limit its ability to capture nonlinear data features. Thus, even with regularization, the RR model does not offer significant advantages in complex data environments, particularly those involving high nonlinearity and interaction effects.

In contrast, the RFR model outperformed both the MLR and RR models on both the training and test sets, achieving a training RRMSE of 1.46% and a test RRMSE of 3.37%. The strength of the RFR model lies in its ensemble learning approach. By constructing multiple decision trees and combining their predictions, RFR effectively captures nonlinear relationships and higher-order interactions within the data. This is particularly beneficial for predicting maize water content, where feature relationships are complex. Through the ensemble of trees, RFR not only improves accuracy but also avoids the overfitting that can occur with a single decision tree. Additionally, the RFR model demonstrates high robustness to noise and outliers, enhancing stability on complex, variable datasets and improving predictive accuracy. Following PSO, the RFR model’s prediction accuracy improved further. The RRMSE for the training and test sets decreased to 1.19% and 3.36%, respectively. PSO optimization fine-tuned the model’s hyperparameters (such as the number of trees and their depth), enabling the RFR model to better align with the data’s distribution and variability. This optimization process enhanced the model’s ability to detect complex patterns, further improving its predictive performance. The successful application of PSO underscores the importance of hyperparameter adjustment in enhancing RFR’s predictive accuracy, particularly when addressing complex nonlinear issues.

In conclusion, while the MLR and RR models perform well for linear relationships, their prediction accuracy is constrained when dealing with complex nonlinear data. In contrast, the RFR model, leveraging the power of ensemble learning, is adept at capturing nonlinear relationships and is more robust to noise and outliers. The PSO optimization further boosted the RFR model’s prediction accuracy by fine-tuning hyperparameters, significantly enhancing its fitting ability and accuracy, thus demonstrating its considerable potential for predicting maize water content.

### 4.2. Impact of Feature Selection and Comparison of Methods

**Figure 9:**
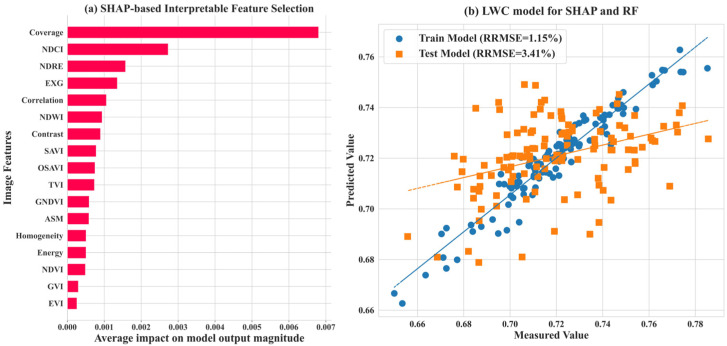
Interpretable feature selection based on SHAP and the random forest algorithm.

Feature selection is critical to the performance of models estimating maize LWC. In this study, feature selection was carried out using PCC + Lasso, resulting in models with RRMSEs of 1.46% and 3.37% for the training and test sets, respectively. These results indicate that this approach effectively reduces feature redundancy while maintaining good generalization performance. However, since Lasso is a linear method, it may fail to capture the contributions of nonlinear variables. To address this, SHAP value analysis (Figure 9a) was used to assess feature importance, and its results were compared with those of Lasso. The SHAP analysis identified Coverage and NDWI as key features in both methods, confirming their pivotal roles in LWC estimation. However, SHAP also highlighted the importance of NDRE and EXG, while Lasso selected Contrast as a less significant feature, suggesting that SHAP is better at identifying more complex feature relationships. The model constructed using features selected by SHAP (Figure 9b) achieved RRMSEs of 1.15% and 3.41% for the training and test sets, respectively. While the training error decreased by 0.31%, the test error increased slightly by 0.04%, indicating that SHAP enhanced the model’s ability to fit training data. However, the features selected by Lasso exhibited more consistent generalization on the test set.

Each method has its strengths and weaknesses. PCC + Lasso, which relies on linear correlations, is suitable for addressing feature collinearity and is computationally efficient with a sparse feature set. However, it struggles with capturing nonlinear relationships and may overlook important variables. SHAP, by assigning contribution values based on model output, offers more intuitive interpretations and better captures nonlinear influences. However, SHAP is dependent on the model’s output, and different model architectures may lead to variations in the selected features. Moreover, SHAP requires more computational resources, posing challenges for large datasets. Thus, in this study, SHAP was primarily used for feature impact analysis rather than feature selection itself.

Given these findings, the study suggests that combining different feature selection methods could enhance the robustness of LWC estimation models. PCC + Lasso can reduce feature redundancy and ensure good generalization, while SHAP provides deeper insights into feature relationships and identifies potential nonlinearities. Future research could explore using Lasso for initial screening, SHAP for optimization, and incorporating other techniques such as Bayesian Optimization, Mutual Information, and XGBoost’s built-in feature importance to improve crop water estimation accuracy, thereby providing more reliable support for UAV-based remote sensing in precision agriculture.

### 4.3. Comparison of ResNet50 Feature Optimization in LWC Models Across Different Growth Stages

**Figure 10:**
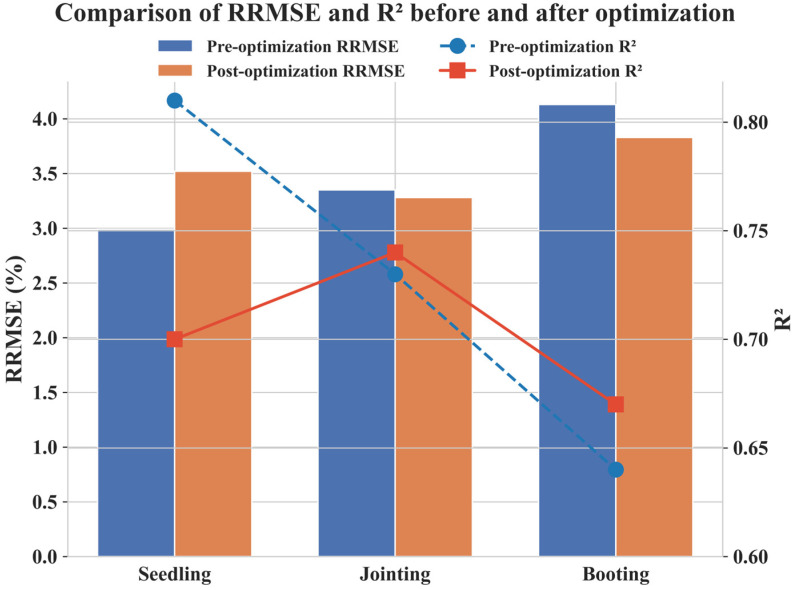
Comparison of maize leaf water content estimation at different growth stages before and after optimization using ResNet50-derived convolutional features.

The LWC estimation model developed in this study exhibited notable variations in performance across different growth stages (Figure 10). The highest error was observed during the booting stage (RRMSE = 4.13%, R^2^ = 0.64), while the jointing stage showed relatively higher accuracy. However, after optimization, model accuracy declined during the seedling stage, with an increase in error. The accuracy of the model is influenced by factors such as canopy structure, water dynamics, and spectral response characteristics. In particular, the increased number of leaf layers during the booting stage enhances spectral mixing effects, thereby increasing the complexity of LWC estimation.

To address the limitations of traditional feature selection methods, this study incorporated convolutional features extracted using the deep learning model ResNet50, in combination with conventional spectral and texture features, to optimize the model (Figure 10). After optimization, the model’s performance improved during the jointing stage (RRMSE = 3.28%, R^2^ = 0.74) and the booting stage (RRMSE = 3.83%, R^2^ = 0.67), indicating that deep learning features enhance LWC estimation under complex canopy structures. However, during the seedling stage, optimization led to an increase in RRMSE (3.52%) and a decline in R^2^, suggesting that ResNet50 has limited applicability at this early stage. Several factors may contribute to the decline in model accuracy during the seedling stage: (1) Soil background interference: Due to insufficient plant coverage, a significant proportion of the spectral signal originates from the soil. ResNet50 may fail to effectively distinguish between soil and vegetation, leading to confusion in water content estimation and reduced model accuracy. (2) Feature redundancy and noise: At the seedling stage, the canopy is relatively simple, and conventional spectral indices may already provide sufficient information. The use of ResNet50 may introduce redundant or noisy features, reducing model robustness and leading to a decline in R^2^. (3) Weak spectral response of leaf water content: As leaf water variation is minimal at this stage, spectral features exhibit low sensitivity to LWC. Consequently, deep learning fails to effectively capture relevant patterns and may even introduce errors. (4) Environmental disturbances: The small plant size makes LWC estimation susceptible to variations in illumination angles and soil moisture, which affect UAV imagery. This results in unstable feature extraction by ResNet50, limiting its optimization effectiveness. In contrast, during the jointing and booting stages, RRMSE decreased and R^2^ improved after optimization, demonstrating that deep learning features are more effective under complex canopy conditions. The contribution of deep learning is particularly significant at stages where spectral signals are heavily influenced by canopy structure.

Overall, ResNet50 optimization improved LWC estimation performance during the jointing and booting stages but exhibited limitations during the seedling stage. Future research could refine feature extraction strategies for early growth stages by: (1) integrating environmental variables (e.g., soil moisture and meteorological data) to reduce soil background interference, (2) incorporating an attention mechanism to enhance sensitivity to key spectral features while suppressing redundant information, and (3) adopting shallow feature extraction strategies to minimize noise and improve model robustness during early growth stages.

### 4.4. Comparative Analysis of Optimization Algorithms

Following PSO optimization, the RFR model’s RRMSE decreased to 1.19% and 3.36% in the training and test sets, respectively, while R^2^ improved to 0.99 (training set) and 0.89 (test set), significantly enhancing the model’s fitting capability. However, the reduction in test error was minimal, decreasing only from 3.37% to 3.36%, suggesting that PSO primarily improved training accuracy while providing limited enhancements to generalization ability. This limitation likely arises from PSO’s tendency toward local search, which fine-tunes the model for training data but reduces adaptability to unseen test data. Despite PSO’s strong performance during training, its generalization capacity remains a challenge, highlighting the need for optimization strategies with stronger global search capabilities.

**Figure 11:**
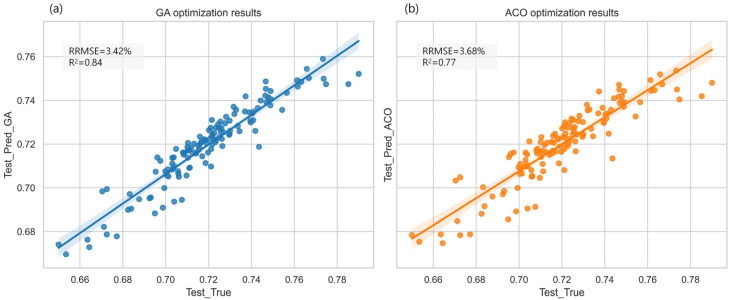
Comparison of Prediction Accuracy Between GA- and ACO-Optimized Models. The solid lines (blue and orange) depict prediction trends, and the shaded areas (blue for GA, orange for ACO) represent the 95% confidence interval, indicating prediction uncertainty.

In comparison, Genetic Algorithm (GA) and Ant Colony Optimization (ACO) exhibit distinct characteristics when optimizing the RFR model (see Figure 11). The GA-optimized RFR model achieved an R^2^ of 0.84 and an RRMSE of 3.42% on the test set. While GA did not surpass PSO in test-set prediction accuracy, it demonstrated improved generalization performance. Unlike PSO, GA employs a global search strategy that mitigates overfitting, resulting in more stable test-set performance. However, GA requires a greater number of evolutionary generations to converge to an optimal solution, leading to higher computational costs than PSO. Additionally, the optimization effectiveness of GA is influenced by selection, crossover, and mutation strategies, which may introduce variability across different datasets. ACO, which simulates the foraging behavior of ants and employs a pheromone update mechanism for parameter optimization, offers advantages in local search efficiency. In this study, the ACO-optimized RFR model achieved an R^2^ of 0.77 and an RRMSE of 3.68% on the test set, exhibiting slightly weaker optimization performance compared to GA and PSO. This outcome may be attributed to ACO’s strong reliance on local optima, which can limit its global search capability. Moreover, ACO’s pheromone update mechanism may introduce instability across different datasets, leading to variations in predictive performance. Nonetheless, ACO demonstrates superior computational efficiency compared to GA, enabling faster convergence to suboptimal solutions and reducing computational costs, making it a viable option when computational resources are constrained.

Overall, PSO achieves the highest training accuracy but has limited generalization ability; GA provides greater test-set stability but incurs higher computational costs; ACO offers enhanced computational efficiency but exhibits weaker global search capability. Future research could explore hybrid optimization strategies, such as integrating PSO’s rapid convergence with GA’s global search capability, to further enhance model predictive performance and improve generalization across diverse environmental conditions.

## 5. Conclusions

This study presents a method for estimating maize leaf water content (LWC) based on UAV multispectral images and a Random Forest Regression (RFR) model. The method integrates vegetation indices, texture features, and image coverage data, utilizing Multivariate Linear Regression (MLR), Ridge Regression (RR), and RFR algorithms for model training. The results demonstrate that the RFR model delivers the best performance for LWC estimation, particularly during the seedling stage, where the RRMSE is 2.99%. However, during the booting stage, the RRMSE increases to 4.13%. Following Particle Swarm Optimization (PSO), the RFR model exhibits a substantial improvement in training set accuracy, reducing the RRMSE from 1.46% to 1.19%. Additionally, the study reveals significant variations in LWC estimation accuracy across different growth stages. In the seedling stage, water distribution is relatively uniform, enabling stable predictions, while during the booting stage, the increased water demand and uneven distribution lead to reduced estimation accuracy. Further analysis indicates that PCC + Lasso effectively minimizes redundant features while preserving generalization ability, whereas the SHAP method optimizes the model through a more detailed feature analysis, improving training accuracy (with the RRMSE dropping to 1.15%). The inclusion of ResNet50 deep learning features significantly enhanced model accuracy during the seedling and jointing stages (RRMSE of 3.52% and 3.28%, respectively), although the improvement during the booting stage was limited (RRMSE decreased from 4.13% to 3.83%). In conclusion, the maize LWC estimation method proposed in this study offers an efficient and accurate approach for crop water management and precision agriculture. It is applicable across various growth stages and water conditions and provides a valuable reference for moisture estimation in other crops.

## Figures and Tables

**Table 1:** Definitions of the vegetation indices extracted from orthorectified multispectral images.

Vegetation Index	Definition	Reference
Excess Green Index (EXG)	EXG = 2G−R−B	[24]
Normalized Difference Vegetation Index (NDVI)	NDVI = $\left(\mathrm{NIR}-R\right)/\left(\mathrm{NIR}+R\right)$	[25]
Normalized Difference Water Index (NDWI)	NDWI = $\left(G-NIR\right)/\left(G+\mathrm{NIR}\right)$	[26]
Normalized Difference Red-Edge (NDRE)	NDRE = (NIR−RE)/(NIR+RE)	[27]
Green Vegetation Index (GVI)	GVI = (2 × NIR−R)/(2 × NIR+R)	[28]
Soil-Adjusted Vegetation Index (SAVI)	SAVI = $\left(\mathrm{NIR}-R\right)/\left(\mathrm{NIR}+R+0.5\right)\;\times \;(1.5)$	[29]
Enhanced Vegetation Index (EVI)	EVI = $\left(\mathrm{NIR}-R\right)/(1+\mathrm{NIR}-2.4\;\times \;R)\;\times \;(2.5)$	[30]
Green-Normalized Difference Vegetation Index (GNDVI)	GNDVI = $\left(\mathrm{NIR}-G\right)/\left(\mathrm{NIR}+G\right)$	[31]
Optimized Soil Adjusted Vegetation Index (OSAVI)	OSAVI = $\left(\mathrm{NIR}-R\right)/(\mathrm{NIR}-R+0.16)$	[29]
Triangular Vegetation Index (TVI)	TVI = $\sqrt{\mathrm{NDVI}+0.5}$	[32]
Normalized Difference Chlorophyll Index (NDCI)	NDCI = (RE−NIR)/(RE+NIR)	[33]

Note: r, g, and b are the DN values normalized to the R, G, and B channels. DN: digital number; CI: color index; RGB: red, green, and blue.

**Table 2:** Descriptive statistics of field-measured maize leaf water content (LNC, Units: %).

Stage	BBCH	Avg.	Max	Min	STDEV	CV	Med.	Skewness	Sample Size
Seedling	12	76.9	88.2	69.6	2.27	3.14	77.2	0.34	90
Jointing	31	67.5	72.6	60.8	2.49	3.46	67.1	0.15	90
Booting	45	75.9	80.3	60.9	2.98	4.16	75.0	0.20	90

Note: BBCH represents Biologische Bundesanstalt, Bundessortenamt und CHemische Industrie. Avg. represents the mean percentage of LWC, while Max and Min denote the highest and lowest values, respectively; STDEV indicates the variability in LWC data, with CV calculated as the ratio of STDEV to the mean. Med. reflects the 50th percentile, and Skewness measures the asymmetry of the data distribution. The sample size refers to the number of data points collected for each growth stage.

## Data Availability

The original contributions presented in this study are included in the article; further inquiries can be directed to the corresponding author.

## References

[1] Wang R.M., He N., Li S.G., Li X., Li M.X. (2021). Spatial variation and mechanisms of leaf water content in grassland plants at the biome scale: Evidence from three comparative transects. Sci. Rep..

[2] Trifilò P., Abate E., Petruzzellis F., Azzarà M., Nardini A. (2023). Critical water contents at leaf, stem and root level leading to irreversible drought-induced damage in two woody and one herbaceous species. Plant Cell Environ..

[3] Song X.Y., Zhou G.S., He Q.J. (2021). Critical Leaf Water Content for Maize Photosynthesis under Drought Stress and Its Response to Rewatering. Sustainability.

[4] Yang F.F., Liu T., Wang Q.Y., Du M.Z., Yang T.L., Liu D.Z., Li S.J., Liu S.P. (2021). Rapid determination of leaf water content for monitoring waterlogging in winter wheat based on hyperspectral parameters. J. Integr. Agric..

[5] Li B., Zhang X., Wang R., Mei Y., Ma J.J. (2021). Leaf water status monitoring by scattering effects at terahertz frequencies. Spectrochim. Acta Part A Mol. Biomol. Spectrosc..

[6] Rezaei M., Ebrahimi E., Naseh S., Mohajerpour M. (2012). A new 1.4-GHz soil moisture sensor. Measurement.

[7] Deng X., Gu H.N., Yang L., Lyu H.F., Cheng Y.G., Pan L.P., Fu Z.J., Cui L.Q., Zhang L. (2020). A method of electrical conductivity compensation in a low-cost soil moisture sensing measurement based on capacitance. Measurement.

[8] Phang S.K., Chiang T., Happonen A., Chang M. (2023). From Satellite to UAV-Based Remote Sensing: A Review on Precision Agriculture. IEEE Access.

[9] Attard M., Phillips R.A., Bowler E., Clarke P.J., Cubaynes H., Johnston D.W., Fretwell P.T. (2024). Review of Satellite Remote Sensing and Unoccupied Aircraft Systems for Counting Wildlife on Land. Remote Sens..

[10] Zhang Z.X., Zhu L.X. (2023). A Review on Unmanned Aerial Vehicle Remote Sensing: Platforms, Sensors, Data Processing Methods, and Applications. Drones.

[11] Zhang P.P., Zhou X.X., Wang Z.X., Mao W., Li W.X., Yun F., Guo W.S., Tan C.W. (2020). Using HJ-CC D image and PLS algorithm to estimate the yield of field-grown winter wheat. Sci. Rep..

[12] Jeong S., Ko J., Choi J., Xue W., Yeom J.M. (2018). Application of an unmanned aerial system for monitoring paddy productivity using the GRAMI-rice model. Int. J. Remote Sens..

[13] Liu T., Wang J.L., Wang J.Y., Zhao Y.Y., Wang H., Zhang W.J., Yao Z.S., Liu S.P., Zhong X.C., Sun C.M. (2025). Research on the estimation of wheat AGB at the entire growth stage based on improved convolutional features. J. Integr. Agric..

[14] Zhang M.N., Zhou J.F., Sudduth K.A., Kitchen N.R. (2020). Estimation of maize yield and effects of variable-rate nitrogen application using UAV-based RGB imagery. Biosyst. Eng..

[15] Zhang Y., Wu J.B., Wang A.Z. (2022). Comparison of various approaches for estimating leaf water content and stomatal conductance in different plant species using hyperspectral data. Ecol. Indic..

[16] Wang X.P., Zhao C.Y., Guo N., Li Y.H., Jian S.Q., Yu K. (2015). Determining the Canopy Water Stress for Spring Wheat Using Canopy Hyperspectral Reflectance Data in Loess Plateau Semiarid Regions. Spectrosc Lett..

[17] Chen S.B., Chen Y.W., Chen J.Y., Zhang Z.T., Fu Q.P., Bian J., Cui T., Ma Y.Z. (2020). Retrieval of cotton plant water content by UAV-based vegetation supply water index (VSWI). Int. J. Remote Sens..

[18] Yang N., Zhang Z.T., Ding B.B., Wang T.Y., Zhang J.R., Liu C., Zhang Q.Y., Zuo X.Y., Chen J.Y., Cui N.B. (2024). Evaluation of winter-wheat water stress with UAV-based multispectral data and ensemble learning method. Plant Soil.

[19] Shu M.Y., Dong Q.Z., Fei S.P., Yang X.H., Zhu J.Y., Meng L., Li B.G., Ma Y.T. (2022). Improved estimation of canopy water status in maize using UAV-based digital and hyperspectral images. Comput. Electron. Agric..

[20] Cheng T., Rivard B., Sánchez-Azofeifa A.G., Féret J.B., Jacquemoud S., Ustin S.L. (2012). Predicting leaf gravimetric water content from foliar reflectance across a range of plant species using continuous wavelet analysis. J. Plant Physiol..

[21] Ndlovu H.S., Odindi J., Sibanda M., Mutanga O., Clulow A., Chimonyo V., Mabhaudhi T. (2021). A Comparative Estimation of Maize Leaf Water Content Using Machine Learning Techniques and Unmanned Aerial Vehicle (UAV)-Based Proximal and Remotely Sensed Data. Remote Sens..

[22] Niu Y.X., Han W.T., Zhang H.H., Zhang L.Y., Chen H.P. (2021). Estimating fractional vegetation cover of maize under water stress from UAV multispectral imagery using machine learning algorithms. Comput. Electron. Agric..

[23] Wang S., Ning Y.F., Shi H.M. (2021). A new uncertain linear regression model based on equation deformation. Soft Comput..

[24] Wang J.L., Chen C., Wang J.C., Yao Z.S., Wang Y., Zhao Y.Y., Wu F., Han D.W., Yang G.S., Liu T. (2025). NDVI Estimation Throughout the Whole Growth Period of Multi-Crops Using RGB Images and Deep Learning. Agronomy.

[25] Stamford J.D., Vialet-Chabrand S., Cameron I., Lawson T. (2023). Development of an accurate low cost NDVI imaging system for assessing plant health. Plant Methods.

[26] Xu H.Q. (2006). Modification of normalised difference water index (NDWI) to enhance open water features in remotely sensed imagery. Int. J. Remote Sens..

[27] Li F., Miao Y.X., Feng G.H., Yuan F., Yue S.C., Gao X.W., Liu Y.Q., Liu B., Ustine S.L., Chen X.P. (2014). Improving estimation of summer maize nitrogen status with red edge-based spectral vegetation indices. Field Crops Res..

[28] Jiang L., Kogan F.N., Guo W., Tarpley J.D., Mitchell K.E., Ek M.B., Tian Y.H., Zheng W.Z., Zou C.Z., Ramsay B.H. (2010). Real-time weekly global green vegetation fraction derived from advanced very high resolution radiometer-based NOAA operational global vegetation index (GVI) system. J. Geophys. Res. Atmos..

[29] Xue J.R., Su B.F. (2017). Significant Remote Sensing Vegetation Indices: A Review of Developments and Applications. J. Sens..

[30] Huete A., Didan K., Miura T., Rodriguez E.P., Gao X., Ferreira L.G. (2002). Overview of the radiometric and biophysical performance of the MODIS vegetation indices. Remote Sens. Environ..

[31] Haralick R.M., Shanmugam K., Dinstein I.H. (1973). Textural features for image classification. IEEE Trans. Syst. Man Cybern..

[32] Gitelson A.A., Kaufman Y.J., Stark R., Rundquist D. (2002). Novel algorithms for remote estimation of vegetation fraction. Remote Sens. Environ..

[33] Broge N.H., Leblanc E. (2001). Comparing prediction power and stability of broadband and hyperspectral vegetation indices for estimation of green leaf area index and canopy chlorophyll density. Remote Sens. Environ..

[34] Mishra S., Mishra D.R. (2012). Normalized difference chlorophyll index: A novel model for remote estimation of chlorophyll-a concentration in turbid productive waters. Remote Sens. Environ..

[35] Mu S.J., Yang H.F., Li J.L., Chen Y.Z., Gang C.C., Zhou W., Ju W.M. (2013). Spatio-temporal dynamics of vegetation coverage and its relationship with climate factors in Inner Mongolia, China. J. Geogr. Sci..

[36] Lee H., Wang J.F., Leblon B. (2020). Using Linear Regression, Random Forests, and Support Vector Machine with Unmanned Aerial Vehicle Multispectral Images to Predict Canopy Nitrogen Weight in Corn. Remote Sens..

[37] Yasin A., Amin M., Qasim M., Muse A.H., Soliman A.B. (2022). More on the Ridge Parameter Estimators for the Gamma Ridge Regression Model: Simulation and Applications. Math. Probl. Eng..

[38] Ali M., Prasad R., Xiang Y., Yaseen Z.M. (2020). Complete ensemble empirical mode decomposition hybridized with random forest and kernel ridge regression model for monthly rainfall forecasts. J. Hydrol..

[39] Chen W.N., Zhang J., Chung H., Zhong W.L., Wu W.G., Shi Y.H. (2010). A Novel Set-Based Particle Swarm Optimization Method for Discrete Optimization Problems. IEEE Trans. Evol. Comput..

[40] Liu T., Yang T.L., Zhu S.L., Mou N.N., Zhang W.J., Wu W., Zhao Y.Y., Yao Z.S., Sun J.J., Chen C. (2024). Estimation of wheat biomass based on phenological identification and spectral response. Comput. Electron. Agric..

